# Clinical Features and Diagnosis of Cardiac Sarcoidosis

**DOI:** 10.3390/jcm10091941

**Published:** 2021-05-01

**Authors:** Claudio Tana, Cesare Mantini, Iginio Donatiello, Luciano Mucci, Marco Tana, Fabrizio Ricci, Francesco Cipollone, Maria Adele Giamberardino

**Affiliations:** 1 COVID-19 Medicine Unit and Geriatrics Clinic, SS Annunziata Hospital of Chieti, 66100 Chieti, Italy; fcipollone@unich.it (F.C.); mag@unich.it (M.A.G.); 2 Department of Neuroscience, Imaging and Clinical Sciences, Institute of Radiology, “SS Annunziata” Hospital, “G. d’Annunzio” University, 66100 Chieti, Italy; cesare.mantini@gmail.com (C.M.); fabrizioricci@hotmail.it (F.R.); 3 Internal Medicine Unit, University Hospital of Salerno, 84121 Salerno, Italy; iginiodonatiello@gmail.com; 4 Internal Medicine Unit, Hospital of Fano, Azienda Ospedaliera Ospedali Riuniti Marche, 61032 Fano, Italy; luciano_mucci@libero.it; 5 2nd Medicine Unit and Department of Vascular Medicine and Cardiovascular Ultrasound, SS Annunziata Hospital of Chieti, 66100 Chieti, Italy; marco_tana@yahoo.it; 6 Department of Medicine and Science of Aging, and CAST, G D’Annunzio University of Chieti, 66100 Chieti, Italy; fcipollone@unich.it

**Keywords:** cardiac sarcoidosis, imaging, biopsy, magnetic resonance imaging, 18F-FDG PET

## Abstract

Cardiac sarcoidosis (CS) is an unusual, but potentially harmful, manifestation of systemic sarcoidosis (SA), a chronic disease characterized by organ involvement from noncaseating and nonnecrotizing granulomas. Lungs and intrathoracic lymph nodes are usually the sites that are most frequently affected, but no organ is spared and CS can affect a variable portion of SA patients, up to 25% from post-mortem studies. The cardiovascular involvement is usually associated with a bad prognosis and is responsible for the major cause of death and complications, particularly in African American patients. Furthermore, the diagnosis is often complicated by the occurrence of non-specific clinical manifestations, which can mimic the effect of more common heart disorders, and imaging and biopsies are the most valid approach to avoid misdiagnosis. This narrative review summarizes the main clinical features of CS and imaging findings, particularly of CMR and 18-Fluorodeoxyglucose Positron Emission Tomography (18F-FDG PET) that can give the best cost/benefit ratio in terms of the diagnostic approach. Imaging can be very useful in replacing the endomyocardial biopsy in selected cases, to avoid unnecessary, and potentially dangerous, invasive maneuvers.

## 1. Introduction

Sarcoidosis is a chronic disease characterized by organ involvement from noncaseating and non-necrotizing granulomas. Genetic predisposition and environmental risk factors were hypothesized as the main actors of the disease pathogenesis [1]. Lungs and intrathoracic lymph nodes are usually the sites that are most frequently affected [2,3] but no organ is spared as the involvement can be also cardiovascular, gastrointestinal, neurological and of the genitourinary system and skin [4,5,6,7].

The cardiovascular involvement is usually associated with a bad prognosis and is responsible for the major cause of death and complications, particularly in African American patients. The diagnosis is often complicated by the occurrence of non-specific clinical manifestations [8], which can mimic the effect of more common heart disorders, and imaging and biopsies are the most valid approach to avoid misdiagnosis.

This narrative review summarizes the main clinical features of CS and imaging findings, particularly of CMR and 18-Fluorodeoxyglucose Positron Emission Tomography (18F-FDG PET) that can give the best cost/benefit ratio in terms of diagnostic approach. Imaging can be very useful in replacing the endomyocardial biopsy in selected cases, to avoid unnecessary, and potentially dangerous, invasive maneuvers.

A literature search was conducted by searching on public databases (PubMed, Scopus). Search queries were:

Sarcoidosis AND Heart AND magnetic resonance imaging OR 18-Fluorodeoxyglucose Positron Emission Tomography OR hybrid imaging OR endomyocardial biopsy;

Sarcoid lesions AND heart AND magnetic resonance imaging OR 18-Fluorodeoxyglucose Positron Emission Tomography OR hybrid imaging OR endomyocardial biopsy;

Cardiac AND sarcoidosis AND magnetic resonance imaging OR 18-Fluorodeoxyglucose Positron Emission Tomography OR hybrid imaging OR endomyocardial biopsy; 

Cardiac AND sarcoid lesions AND magnetic resonance imaging OR 18-Fluorodeoxyglucose Positron Emission Tomography OR hybrid imaging OR endomyocardial biopsy.

Papers were excluded if they were duplicated or if the topic was not adherent to the theme of cardiac sarcoidosis and magnetic resonance imaging, 18-Fluorodeoxyglucose Positron Emission Tomography, hybrid imaging or endomyocardial biopsy.

### Epidemiology

The real epidemiology of cardiac sarcoidosis remains almost unknown, as clinic findings are often absent or nonspecific. Five percent of patients with pulmonary or systemic sarcoidosis can be characterized by some manifestations of cardiac involvement [9]. The prevalence can reach 25% in autopsy studies [10] and when the MRI is used as a main diagnostic technique for detection [11]. Cardiac sarcoidosis is defined as isolated (without other signs of systemic involvement) in 23–29% of total cases [12,13].

These patients often have worse left systolic ventricular function and a higher incidence of ventricular tachycardia than patients with systemic sarcoidosis [14].

The extent of left ventricular dysfunction is the most important predictor of overall survival [15], and the high mortality and morbidity of cardiac involvement in sarcoidosis requires a rapid diagnosis and a prompt treatment of the condition, to avoid deterioration that can quickly become irreversible [16].

The median age of onset of cardiac sarcoidosis is 50 years [12]. Thanks to the evolution of the modern noninvasive diagnostic techniques, the detection rate has increased over the last 25 years [12].

A significant predictor of in-hospital mortality is represented by the black race, which has been associated with an increased risk of 21% (odds ratio (OR), 1.21; 95% confidence interval-CI-1.16–1.27 (*p* < 0.001)). In these patients, the most prevalent CV manifestations are heart failure (≈16%) and arrhythmias (≈15%), while implantable cardioverter-defibrillator placement rates were ≈7.5 per 1000 sarcoidosis hospitalizations (Ptrend = 0.95) [17].

## 2. Clinical Features of Cardiac Sarcoidosis

Clinical manifestations of cardiac sarcoidosis are correlated with the presence of typical noncaseating granulomas and inflammatory processes involving cytokine release by type 1 helper T (Th1) cells. Specifically, a high expression of Il-1 alpha, Il-2, Il-12 (localized in multinucleated giant cells and macrophages of granulomas) and IFN-gamma (in lymphocytes and endothelial vascular cells) was found in the myocardial tissue of patients with cardiac sarcoidosis [18]. The most frequent clinical symptoms are palpitations, lipothymia and the occurrence of syncope. The occurrence of sudden cardiac death is linked to arrhythmias associated with granulomas and subsequent scarring phenomena, possible foci of ectopic conduction and interruption of normal conduction or recovery pathways. Asthenia, dyspnoea and orthopnea are related to heart failure, less commonly to arrhythmias in cardiac sarcoidosis, and the involvement of the ventricle can lead to the onset of cardiomyopathy at a young age [19].

Among all arrhythmias, the most represented are those secondary to alterations of the conduction pathways (atrioventricular node, bundle of His and intraventricular pathways), which can progress over time as the disease evolves. According to a large population study, atrioventricular block is the most common clinical manifestation of the disease onset [20], presenting in 44% of patients with cardiac sarcoidosis [17]. The progression, even silent, towards a complete atrioventricular block can lead to clinical symptoms such as syncope, sudden death and also to ventricular tachyarrhythmias [20].

Ventricular tachyarrhythmias represent the second most common clinical manifestation of the disease onset, affecting approximately 30% of patients [12]. These include a broad spectrum of electrophysiological and clinical conditions, ranging from ventricular extrasystoles to ventricular fibrillation. Acute inflammatory mechanisms with secondary scarring and fibrotic events can determine anomalous reentry pathways based on ventricular tachyarrhythmias [21]. This hypothesis is further supported by the demonstration that ventricular tachyarrhythmias occur frequently in the first year after the introduction of corticosteroid therapy, when the inflammatory picture may not yet be completely controlled [22]. It should be emphasized that ventricular tachyarrhythmias are an independent predictor of mortality in patients with cardiac sarcoidosis [23] and require ICD implantation more frequently than in patients with dilated cardiomyopathy [24]. Sudden cardiac death is the leading cause of death related to sarcoidosis in Japan, while in America it is the second, preceded by pulmonary complications [25]. The real incidence is unknown, although it was estimated that it can occur in 14% of patients from observational studies [26]. The main mechanisms can be represented by the progression of conduction blocks or ventricular tachyarrhythmias related to the presence of granulomas. The presence of granulomas of the left ventricle was demonstrated in almost all cases, and of the interventricular septum in 94% of cases [16].

Supraventricular tachyarrhythmias have a prevalence between 19% and 32% [27,28], three times greater than in sarcoidosis patients without cardiac involvement [29]. The most frequent supraventricular tachyarrhythmia is atrial fibrillation, followed by atrial tachycardia and atrial flutter; almost all of these patients are symptomatic and the presence of the left atrial enlargement increases the risk of supraventricular arrhythmias about six times [28].

Heart failure is an onset manifestation of the disease in 16% of cases, less frequently than arrhythmias [30]. Despite this, heart failure represents one of the most important predicting factors of mortality according to the severity based on clinical NYHA classification, and instrumental (end-diastolic diameter of the left ventricle) data [23], and it is the second cause of death, only after sudden death from ventricular arrhythmias [25]. Heart failure can be caused by secondary pulmonary hypertension and/or cardiac remodeling caused by the inflammatory and scarring processes typical of the disease, leading to diastolic or systolic dysfunction.

Patients with sarcoidosis also have a 1.65 increased risk of cardiovascular events compared to the control population, after the correction for risk factors [31]. A causal connection could also be represented by the occurrence of inflammatory processes underlying both atherosclerosis and sarcoidosis. Less common mechanisms can be represented by myocardial ischemia associated with vasculitis of the coronary arteries, which are reported very rarely in the literature [32].

## 3. Diagnosis of Cardiac Sarcoidosis

The diagnostic criteria of cardiac sarcoidosis were presented by many international scientific societies and an optimal diagnostic algorithm is still under discussion.

The JMHW guidelines of 2006 [33] have suggested that a diagnosis of cardiac sarcoidosis can be reached by (1) histological demonstration of the presence of noncaseating granulomas in the myocardium in a patient with a histological or clinical diagnosis of sarcoidosis in other organs or tissues, and (2) a clinical or histological diagnosis of extra-cardiac sarcoidosis in association with at least three major cardiac criteria: advanced atrioventricular block (AVB), basal interventricular septal thickening and cardiac uptake of gallium-67, left ventricular ejection fraction is less than 50% for four minor criteria: (1) ECG abnormalities (ventricular extrasystoles, ventricular tachycardia, right bundle branch block, Q wave abnormalities and axial deviation on the standard electrocardiogram); (2) echocardiographic abnormalities (segmental-type morphological or wall mobility abnormalities, ventricular aneurysms, or wall thickening); (3) perfusion defects revealed by thallium or technetium scintigraphy; (4) late gadolinium reinforcement on cardiovascular magnetic resonance imaging; (5) diffuse infiltration or interstitial fibrosis in the myocardium on the biopsy.

The JMHW guidelines emphasize the importance of an endomyocardial biopsy to demonstrate cardiac involvement by sarcoid tissue. However, this approach has the great limitation of being extremely invasive in the clinical practice. Furthermore, myocardial involvement in cardiac sarcoidosis is patchy and multifocal and, when combined with the limitations of current sampling techniques, many patients can have nondiagnostic biopsies [34]. Despite its high specificity, therefore, an endomyocardial biopsy can have a low sensitivity for the diagnosis of cardiac sarcoidosis. Another limit of the JMH guidelines is that the uptake of Gallium-67 (67 Ga), still considered among the diagnostic criteria, is not currently used in most centers due to its limited diagnostic accuracy, as demonstrated by several studies [34,35,36,37].

In the latest update, fatal ventricular arrhythmias (e.g., sustained ventricular tachycardia and ventricular fibrillation) and anatomic abnormalities of the ventricular wall (e.g., aneurysm of the ventricular wall, midbasal septum thickening) were included in the major criteria, and diagnostic methods such as the 18F-FDG PET and CMR were included in the major criteria as diagnostic tools [38].

The consensus statement from the Heart Rhythm Society (HRS), in association with the American College of Chest Physicians (ACCP), the American Heart Association (AHA), the Asia Pacific Heart Rhythm Society (APHRS), the European Heart Rhythm Association (EHRA), and the World Association of Sarcoidosis and other Granulomatous Disorders (WASOG) in 2014 recognized both histological (defined) and clinical (probable) criteria for the diagnosis of cardiac sarcoidosis. These guidelines have included both the use of LGE-CMR and 18F-FDG PET in the diagnostic criteria [39].

### 3.1. Patient Screening for Cardiac Involvement

The first evaluation of patients with suspected CS should include a baseline ECG, which is useful to reveal the most common alterations. A normal ECG, however, does not exclude the presence of even minimal cardiac involvement, but is most often altered in overt CS. Echocardiography is important to reveal the most frequent, although nonspecific, abnormalities such as segmental-type morphological or wall-thickening wall hypomobility abnormalities. Such findings, however, can be found in other cardiac disorders, and are not specific to CS, but can be useful for the first screening of the severity of the disease [33].

Some abnormalities such as the cardiac right ventricular involvement can lead to the misdiagnosis of arrhythmogenic right ventricular cardiomyopathy (ARVC). The 2010 ARVC task force criteria failed to differentiate between CS and hereditary ARVC. Some authors have recently demonstrated how prolonged PR interval, advanced AVB, long duration of QRS associated with reduced LVEF, right involvement of the ventricular apex and positive findings on the 18F-FDG PET should be considered as suspected for CS [40].

### 3.2. Cardiac MRI

**Figure 1:**
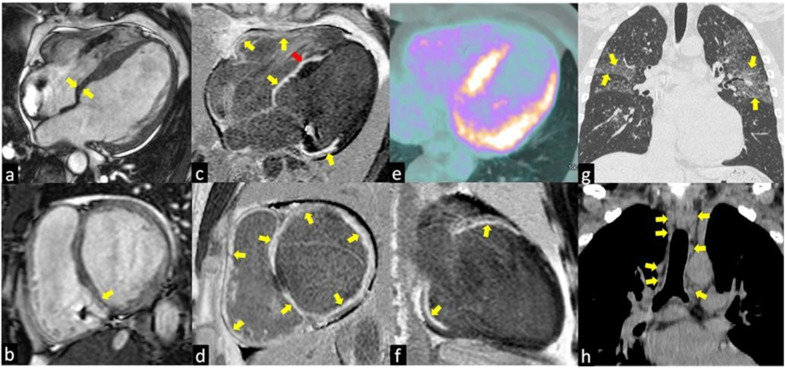
The images show a case of typical Cardiac Sarcoidosis characterized by the presence of LGE of all basal segments of both ventricles (yellow arrows in **c**,**d**,**f**) with the predominantly transmural distribution involving more than one coronary territory and the right ventricular side of the interventricular septum (red arrow in **c**). Bright blood cine sequences show the thinning of the basal septum (yellow arrows in **a**,**b**). Coronal computed tomography (CT) scans show the typical perilymphatic distribution of micronodules with upper lobe predilection (yellow arrows in **g**) and hilar and mediastinal bilateral lymphadenopathy (yellow arrows in **h**). The 18F-fluorodeoxyglucose positron emission tomography (**e**) revealed an increased uptake in the septal and lateral left ventricle myocardial segments in a patient with systemic sarcoidosis.

CMR allows a rapid, accurate and non-invasive evaluation of clinical or sub-clinical cardiac sarcoidosis, thanks to the high spatial and soft-tissue resolution [41]. At present, it is one of the preferred techniques for evaluating cardiac sarcoidosis [42,43], having a high sensitivity and specificity of 75–100% and 76–78%, respectively [35], and thanks to the lack of ionizing radiation. Bright blood cine sequences allow an accurate evaluation of biventricular volume and function, mass and myocardial segment thickness (Figure 1c,d).

**Figure 2:**
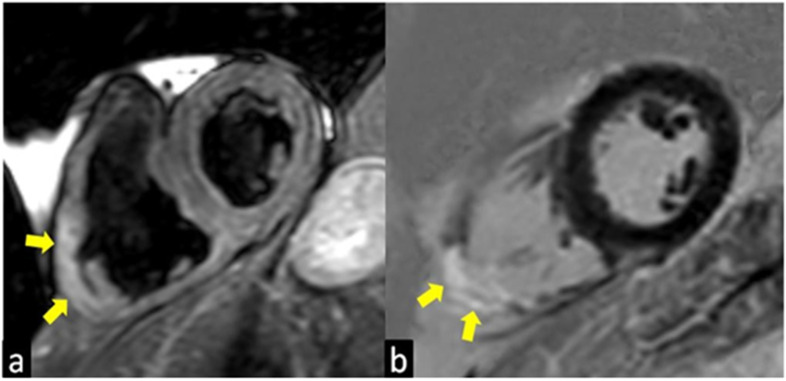
T2-weighted (**a**) and Late Gadolinium Enhancement (LGE; **b**) images show a case of atypical presentation of the acute phase of Cardiac Sarcoidosis, characterized by the presence of edema (yellow arrow in **a**) and LGE (yellow arrow in **b**) of the inferolateral wall of the right ventricle with the transmural distribution.

With the T2-weighted images and the evaluation of early gadolinium uptake, it is possible to reveal respectively the focal presence of acute inflammation (edema) (Figure 2a) and myocardial hyperemia in the thickened myocardium where the granulomas infiltration is located (Figure 2a,b) [44]. Instead, the chronic phase is characterized by ventricular systolic dysfunctions and dilatation often associated with basal septum wall thinning (Figure 1c,d) [45].

Gadolinium is a biologically inert tracer that diffuses freely into the extracellular space, but is unable to cross the intact cell membrane and exhibits a slow washout from damaged cardiomyocytes, identifying areas of myocyte necrosis during the acute phase of CS and areas of macroscopic interstitial fibrosis (scar) during the chronic phase of CS. It is important to underline that late enhancement with gadolinium is not specific for cardiac sarcoidosis but can be observed also in other infiltrative pathologies such as amyloidosis, cardiomyopathies (e.g., hypertrophic cardiomyopathy), myocarditis and ischemic lesions [46].

Typical aspects of cardiac sarcoidosis are: (1) presence of edema (acute phase) and LGE in the basal and mid interventricular septum (Figure 1c,f); (2) presence of both non-ischemic (intramyocardial or subepicardial) (Figure 1e) and ischemic (subendocardial or transmural) (Figure 1f,h) LGE patterns, the latter characterized by the involvement of more than one coronary territories [11]; (3) LGE of the right ventricular side of the interventricular septum (Figure 1e); (4) transmural LGE of thin and akinetic segments in the chronic phase of CS (Figure 1e,f) [46]. The presence of extensive LGE was associated with a poor prognosis by several studies, index of strong sarcoid activity. A meta-analysis including 7 studies and 694 subjects suggested that the presence of LGE among CS patients was associated with an increased risk of cardiovascular death or ventricular arrhythmia [47].

LGE, as well as T1 and T2 mapping techniques, can be used for monitoring the response to anti-inflammatory therapies.

CMR has some advantages over PET imaging, as there is no exposure to ionizing radiation, there is no need for patient preparation such as a specific diet prior to the image acquisition, and it also allows the assessment of cardiovascular morphology, ventricular function, valve and flow quantification [47,48] and the identification of several extracardiac collateral findings [49,50]. However, CMR imaging is limited in patients with pacemakers, or other recently implanted metal devices, and gadolinium is contraindicated in patients with advanced renal disease (estimated glomerular filtration rate [eGFR] < 30 mL/min) [51].

## 4. 18F-FDG PET Study of Cardiac Sarcoidosis

The 18F-FDG PET is useful in patients with sarcoidosis as it allows to distinguish normal tissues from sites of active inflammation [52]. This ability derives from the fact that Fludeoxyglucose is a glucose analogue useful for the differentiation between normal tissue and active inflammatory lesions since the activated macrophages within granulomas have a high rate of metabolic activity and glucose utilization [53]. The myocardium is a metabolically active tissue, so careful patient preparation is required before the examination. Several techniques were developed to suppress the physiological absorption of fluorodeoxyglucose by the myocardial tissue to optimize the image [53]. The Joint SNMMI-ASNC Expert Consensus paper recommends at least two high-fat meals (>35 g) and low carbohydrate content (<3 g) the day before the exam, followed by fasting. This technique guarantees an uptake suppression in 91% of cases [54,55]. A recent work reports a suppression of uptake in 95% of cases on a high-fat, low-carbohydrate diet 36 h before the examination, followed by intravenous unfractionated heparin [56]. The 18F-FDG PET can identify active cardiac sarcoidosis with a high sensitivity (Figure 1e) [57]. The joint analysis of the results of seven studies involving 164 patients, in which the 18F-FDG PET was compared with the standard criteria, showed a sensitivity of 90% and a specificity of 78% [57]. Similar results were obtained from a recent meta-analysis by Kim et al., which found sensitivity and specificity values of 84% of 83%, respectively [58]. Specificity increases with the presence of extra-cardiac findings at total body 18F-FDG PET, such as characteristic mediastinal and/or hilar lymphadenopathy.

The 18F-FDG PET is more sensitive than gallium or thallium scintigraphy and technetium SPECT [59]. Some studies also suggest that the 18F-FDG PET can sometimes have higher diagnostic accuracy than standard clinical criteria and MRI, both for the diagnosis of cardiac sarcoidosis and for the monitoring of steroid therapy [59,60].

The 18F-FDG PET has proven to be a good technique to follow patients undergoing immunosuppressive treatment and to evaluate the therapy response, with the ability to quantify inflammation using the standardized absorption value (SUV). The 18F-FDG PET can be useful to orient the most appropriate immunosuppressive therapy, the duration of treatment and drug dosage [61,62,63].

The use of the 18F-FDG PET is recommended in combination with myocardial blood flow (MPI) evaluation through PET 13N (ammonia) or PET 82Rb (rubidium) in order to receive information on both the inflammatory and fibrotic components [64]. Four phases of disease can therefore be considered: normal (normal perfusion and normal absorption of 18FDG), early stage (no or slight perfusion defect with a concordant increase in 18FDG absorption), advanced stage (moderate perfusion defect with a corresponding increase in the absorption of 18FDG) and stage of fibrosis (severe perfusion defect with little or no absorption of 18 FDG) [65].

Despite these advantages, the diagnosis of CS with the 18F-FDG PET remains a challenge. Patients may have comorbidities, such as ischemic cardiomyopathy, which can make a diagnosis even more difficult. Scarring areas can be seen in both diseases, and typical inflammation areas of CS can be misdiagnosed as hibernating myocardium. The same can happen in patients with active myocarditis or systemic rheumatological conditions with cardiac involvement [57].

### 4.1. Hybrid PET/CMR Imaging

The combination of PET with CMR in a single acquisition is now possible with the emergence of new hybrid PET-CMR systems, thus the strengths of PET and CMR can be combined. Despite high costs, hybrid scanners can offer great benefits due to the high accuracy and reduced risk of complications [66,67]. The MRI has a high negative predictive value and excellent sensitivity in detecting fibrosis, and the PET is important for its ability to visualize and quantify inflammatory disease activity and extracardiac involvement. In limited studies, the combination of both modalities allows improved detection rates, greater accuracy, and changes in therapeutic decision making [68,69,70]. The association of both techniques can give additional points of evaluation of scar and inflammation findings, being useful for patient management and for the classification of various stages of the disease.

Preparation for hybrid PET/CRM is the same as for FDG-PET. The hybrid approach has the advantage of being able to remove redundant sequences to shorten exam time [71].

### 4.2. Endomyocardial Biopsy

Despite the introduction of advanced imaging techniques, endomyocardial biopsy still represents the “gold standard” for the diagnosis of cardiac sarcoidosis. However, an endomyocardial biopsy is only recommended in patients in whom it is not possible to obtain histological confirmation of noncaseating granulomas in other organs or tissues. This is because the endomyocardial biopsies have a high procedural risk and low sensitivity (<25%) in the identification of noncaseating granulomas, first because the areas that are most frequently affected are the basal septum and lateral wall of the left ventricle, which are difficult to access for biopsy, and second because often the myocardial infiltration is focal and irregular [72]. If needed, an endomyocardial biopsy can be performed under the guidance of an electroanatomical mapping or advanced imaging techniques such as MRI [73].

## 5. Conclusions

As systemic sarcoidosis, CS remains a challenging issue in the matter of diagnostics [74,75]. The evolution of diagnostic techniques in recent years has led to a significant improvement in the detection and classification of the severity of the disease.

Despite the fact that the present gold standard is represented by endomyocardial biopsies, the high invasiveness and risk of false negatives suggests the need for searching and validating new diagnostic algorithms including non invasive methods. As increasing information is available, there is less need for an invasive diagnostic approach to reach a definitive diagnosis of cardiac sarcoidosis. We think that the use of Hybrid PET/CMR imaging could change the global approach to this harmful and complex disease in the future.

## Data Availability

Not applicable.
